# Production of reactive oxygen species by *PuRBOHF* is critical for stone cell development in pear fruit

**DOI:** 10.1038/s41438-021-00674-0

**Published:** 2021-12-01

**Authors:** Xiaoqian Wang, Siqi Liu, Huili Sun, Chunyan Liu, Xinyue Li, Yang Liu, Deguo Lyu, Guodong Du

**Affiliations:** College of Horticulture, Shenyang Agricultural University, Shenyang, 110866 P. R. China

**Keywords:** Secondary metabolism, Plant signalling

## Abstract

The production of reactive oxygen species (ROS) by NADPH oxidase, which is also referred to as respiratory burst oxidase homolog (RBOH), affects several processes in plants. However, the role of RBOHs in cell wall lignification is not well understood. In this study, we show that *PuRBOHF*, an RBOH isoform, plays an important role in secondary wall formation in pear stone cells. ROS were closely associated with lignin deposition and stone cell formation according to microscopy data. In addition, according to the results of an in situ hybridization analysis, the stage-specific expression of *PuRBOHF* was higher in stone cells than in cells of other flesh tissues. Inhibitors of RBOH activity suppressed ROS accumulation and stone cell lignification in pear fruit. Moreover, transient overexpression of *PuRBOHF* caused significant changes in the amount of ROS and lignin that accumulated in pear fruit and flesh calli. We further showed that PuMYB169 regulates *PuRBOHF* expression, while *PuRBOHF*-derived ROS induces the transcription of *PuPOD2* and *PuLAC2*. The findings of this study indicate that *PuRBOHF*-mediated ROS production, which is regulated by a lignin-related transcriptional network, is essential for monolignol polymerization and stone cell formation in pear fruit.

## Introduction

Pear (a member of the *Pyrus* genus), which belongs to the Rosaceae family, is an important fruit tree species and is widely cultivated throughout temperate regions worldwide. At least 22 *Pyrus* species have been identified worldwide^1^, among which five species, *P*. *ussuriensis*, *P*. *pyrifolia*, *P*. *bretschneideri*, *P*. *communis*, and *P*. *sinkiangensis*, are the most cultivated^2^. Unlike the fruit of other *Pyrus* species, *P*. *ussuriensis* fruit has a relatively high stone cell content, which is a crucial factor affecting fruit quality^3^. The presence of stone cells contributes to the rough flesh texture of some pear fruit, thereby reducing their economic value^4^. Therefore, it is essential to reduce the content of stone cells to improve pear quality.

Pear stone cells are sclerenchyma cells formed by the secondary deposition of lignin within the primary walls of parenchyma cells^5^. The formation of stone cells is strongly associated with the synthesis, transfer, and deposition of lignin in pear flesh^6^. The lignin polymers in pear mainly comprise two monolignols, coniferyl, and sinapyl alcohols^7^. As the main building blocks of lignin polymers, monolignols are synthesized via phenylpropanoid- and monolignol-specific pathways^8^. Several enzymes involved in this pathway have been identified^9–11^. Xue et al.^4^ reported that PbrMYB169, a MYB transcription factor, positively regulates monolignol biosynthesis in pear fruit. After monolignols are formed, they are translocated to the cell wall where they are polymerized by peroxidase and laccase^12^. In addition to the lignin monomers, peroxidases, and laccases required to form monolignol radicals, additional substrates such as hydrogen peroxide (H_2_O_2_) and molecular oxygen (O_2_) are required^13^. However, the source of reactive oxygen species (ROS) required for the peroxidase and laccase activity remains unknown.

Although early research focused on the toxicity of ROS, interest has shifted toward their role as signaling molecules in a wide range of physiological processes, such as seed germination, root growth, stomatal opening, pollen tube elongation, and aerenchyma formation^14–20^. ROS signaling also plays an important role in plant lignification. In *Arabidopsis*, the scaffolding of NADPH oxidases to downstream targets of ROS produced might enable localized lignin deposition in the endodermis^21^. Furthermore, exogenous H_2_O_2_ was shown to increase the lignin content in rice roots, whereas diphenyleneiodonium chloride (DPI) inhibited this phenomenon^22^. Heng et al.^23^ found that H_2_O_2_ from polyamine metabolism affects lignification in pear exocarp tissue. However, a molecularly defined signaling pathway through which ROS production in pear fruit in induced during lignification has not been identified.

NADPH oxidases, which are also referred to as respiratory burst oxidase homologs (RBOHs) and are localized in the cell plasma membrane, are the most extensively studied sources of extracellular ROS in plants^24^. In addition to being pivotal for defense against biotic and abiotic stresses, various RBOHs control a large number of developmental processes in response to both internal and external stimuli^25^. In plants, *RBOHs* constitute a multigene family, and each homolog has a specific role. In *Arabidopsis, AtRBOHB* regulates seed after ripening^26^, *AtRBOHC* regulates root hair formation^27^, and *AtRBOHE* regulates anther and pollen development^18^. Moreover, some RBOHs mediate ROS production during lignin production. In pear, three *PbRBOH* components are closely related to known lignification-related *RBOH*s^28^. However, their functionality has yet to be validated.

Lignification is characterized by an increase in ROS production, which might act as a developmental signal in secondary wall differentiation^17^. Although some studies have suggested the involvement of RBOH in the lignification of pear stone cells, it remains unclear which RBOH isoforms are involved and how the ROS-producing system interacts with the lignin-related transcriptional network. This study was conducted to identify the *RBOH* genes involved in stone cell lignification and to clarify the molecular mechanisms underlying ROS signaling pathways. Our results will provide guidance for improving fruit quality through a reduction in the content of stone cells.

## Materials and methods

### Plant materials

Sixty-year-old *P. ussuriensis* ‘Nanguo’ trees in an orchard at Shenyang Agriculture University, Shenyang, China, were used as materials. Fruit samples were collected at 20, 40, 55, 65, 100, and 130 days after full bloom (DAFB). Fresh fruits were used for histochemical analysis, and some fruits were stored at −80 °C for further analysis. Three replicates were included in this study, and each biological replicate comprised ten fruits. Fresh roots, stems, leaves, and flowers were sampled from the same plant.

### Analysis of stone cell and lignin contents

The stone cell content was determined via a frozen HCl treatment as previously described^29^ and was calculated as follows: stone cell content (%) = stone cell dry weight (g DW)/flesh fresh weight (g FW) × 100. The acetyl bromide method was used to estimate the lignin content as described by Anderson et al.^30^. The lignin content was expressed as a percentage and calculated as follows: lignin content (%) = calculated lignin content/calculated DW × 100.

### Lignin histochemical assays

Hand-cut sections were prepared from fresh pear fruit tissues to confirm the presence of stone cell clusters in the fruit flesh. The sections were placed in a phloroglucinol solution for 10 min and then treated with 30% HCl for 5 min. The stained sections were imaged using a hand-held camera. For microscopy analysis, sample preparation, including sample fixing, embedding, and sectioning, was performed as described by Tao et al.^31^. The sections were stained with phloroglucinol-HCl and examined using an optical microscope.

### Determination of the H_2_O_2_ content

The H_2_O_2_ content was determined by measuring the production of H_2_O_2_-titanium complexes formed through the reaction of tissue H_2_O_2_ with titanium tetrachloride^32^. The H_2_O_2_ levels were measured at 415 nm and quantified using a standard curve.

### Histochemical assays for ROS

The H_2_O_2_ level in flesh tissues was monitored using DAB (3,3-diaminobenzidine)-stained sections following the methods described by Choi et al.^33^. Hand-cut fruit sections were subsequently dipped in DAB-HCl solution and incubated in the dark at room temperature. Then, the samples were boiled in 90% ethanol for 10 min for bleaching. The stained sections were subsequently imaged with a hand-held camera. In situ detection of H_2_O_2_ was performed using the fluorescent dye 2′,7′-dichlorodihydrofluorescein diacetate (H_2_DCF-DA) staining method according to the methods of Chen et al.^34^. The pear fruit tissue was cut into 10 μm sections for histological examination using a sliding freezing microtome (CM1850, Leica, Germany) and incubated with a 10 μM solution of H_2_DCF-DA for 5 min in the dark. The accumulation of H_2_O_2_ was observed as green fluorescence using a confocal microscope (TCS SP8, Leica, Germany).

### Transmission electron microscopy

In situ detection of subcellular ROS localization was conducted via transmission electron microscopy with cerium chloride (CeCl_3_) staining. The pear flesh was cut into small (1–2 mm^3^) pieces and then incubated in 50 mM 3-(N-morpholino)propanesulfonic acid (MOPS) containing 5 mM CeCl_3_ at pH 7.2 for 1 h. The samples were then dehydrated and fixed based on the methods of Bestwick et al.^35^.

### Chemical treatments

To evaluate the role of NADPH oxidase as a source of ROS production during lignin biosynthesis, the flesh of Nanguo pear fruit at 40 DAFB were injected with H_2_O_2_ (0.1 ml of 500 μM H_2_O_2_) and diphenyleneiodonium chloride (20 μM DPI; an NADPH oxidase inhibitor) using a syringe without a needle. Pear fruit injected with distilled water served as controls. After 10 days, flesh tissue from the injection sites was collected for histochemical and further analyses.

To examine the response pattern of *PuRBOHF* to various abiotic stresses and hormone treatments, Nanguo pear fruit at 40 DAFB were used. For chilling treatment, the fruits were incubated at 4 °C in a dark growth chamber. For the wounding treatment, scissors were used to make cuts ~0.5 cm in length in the peel. For hormone treatment, the fruits were immersed in abscisic acid (ABA), salicylic acid (SA), and ethephon (ETH) solutions for 15 min. Untreated fruits were used as controls. For the control, wounding, and hormone treatments, the fruits were incubated for 24 h in the dark in a growth chamber at 18 °C and 70% relative humidity. The flesh tissue was then collected for qRT-PCR analysis.

### Measurement of NADPH oxidase activity

Plasma membranes were prepared according to the procedure described by Zhang et al.^36^. The activity of plasma membrane NADPH oxidase was determined based on the reduction of 2,3-bis(2-methoxy-4-nitro-5-sulfophenyl)-2H-tetrazolium-5- carboxanilideinner salt (XTT) by O^2^ − radicals as described by Sagi and Fluhr^37^. The assay reaction media included 50 μl of enzyme extract, 0.5 mM XTT, 0.1 mM NADPH, 2.0 mM CaCl_2_ and 100 mM Tris–HCl (pH 7.5). XTT reduction was monitored at 470 nm in the presence or absence of 50 units of SOD.

### Isolation of developing stone cells from fruit flesh tissues

Stone cells were isolated from flesh tissues at 40 DAFB according to the method of Xue et al.^4^. The frozen flesh samples were thawed in RNase-free water at 4 °C and then incubated on ice for 30 min with periodic vortexing. After centrifugation, the stone cells were resuspended in RNase-free water that included glycine, collected via slow centrifugation, and stored at −80 °C for RNA extraction.

### Expression profiles of genes

Total RNA was extracted using the cetyltrimethylammonium bromide (CTAB)-based method. First-strand cDNA was synthesized using a PrimeScript RT Reagent Kit (Takara, Tokyo, Japan) according to the kit instructions. qRT-PCR was performed on a 7500 Real-time PCR system (Applied Biosystems, Foster City, USA) using a SYBR Green Kit (Takara, Tokyo, Japan). *PuActin* served as the internal control. The primers used are listed in Table S1.

### Subcellular localization

The vector construction and experimental procedures for determining the subcellular location of PuRBOHF were performed according to the methods of Cheng et al.^38^. The *PuRBOHF* coding region was fused in frame to the green fluorescent protein (GFP) N-terminus to form a *CaMV35S-PuRBOHF-GFP* fusion vector. The fusion constructs were transferred into *Agrobacterium tumefaciens* strain GV1301 via the freeze–thaw method. The leaves of *Nicotiana benthamiana* plants were infiltrated through their abaxial surfaces with the *Agrobacterium* suspension. At 72 h post-infiltration, the whole leaf tissues of the infiltration sites were collected, stained with DAPI, and examined using a confocal microscope (TCS SP8, Leica, Germany).

### RNA in situ hybridization

Flesh tissues were collected from pear fruit at 40 DAFB, fixed in formaldehyde solution for 12 h at 4 °C, embedded in paraffin, and cut into 7 µm thick sections. The sections were subsequently hybridized, and probes were detected with NBT (nitro blue tetrazolium chloride) according to a previously described procedure^18^.

### Transient expression of pear fruit and fruit calli

A *pCAMBIA1301S-PuRBOHF* overexpression vector was obtained by cloning the ORF of *PuRBOHF* into a *pCAMBIA1301S* vector. To generate *PuRBOHF*-silenced constructs, a 250-bp fragment of *PuRBOHF* was amplified and cloned into a *pFGC5941* vector. The primers used are shown in Table S2. The constructs were then transformed into *Agrobacterium tumefaciens* strain GV1301 using a previously described method. The transformed *Agrobacterium* cells were then injected into the flesh of Nanguo pear fruit at 40 DAFB using needleless syringes^39^. Thirty fruits were injected with each construct. The injected fruits were left on the tree for 10 days under low-light conditions imposed by shading. Then, the fruits were randomly collected for histochemical analysis and other measurements.

Pear calli were induced from the flesh of young Nanguo fruit according to the methods of Bai et al.^40^. The transformation of pear calli was performed as follows: fruit calli were soaked in *Agrobacterium* suspensions for 10 min. After 3 days of coculture, the calli were screened using hygromycin B on MS solid media in the dark. Then, the lignin content of the transformed calli was determined as previously described.

### GUS analysis

A GUS reporter gene was employed to study the effects of H_2_O_2_ on the expression patterns of the *PuPOD2* and *PuLAC2* genes according to the methods of Wu et al.^41^. The promoters of *PuPOD2* and *PuLAC2* were fused to a *pBI101-GUS* vector to generate the *proPuPOD2*-*GUS* and *proPuLAC2*-*GUS* reporters, respectively. Tobacco leaves were used for transient GUS activity assays. *Agrobacterium tumefaciens* strain GV1301 cells containing each of these two reporters were infiltrated into tobacco leaves (5-week old). For H_2_O_2_ treatment, infiltrated tobacco leaves were sprayed with a 1 mM H_2_O_2_ solution, and those treated with distilled water were used as controls. Histochemical GUS staining was performed as described by He et al.^42^. Fluorometric determination of GUS activity was conducted as previously described^43^.

### Dual-luciferase assays

A dual-luciferase reporter assay was used to verify the interaction between PuMYB169 and the *PuRBOHF* promoter^44^. To generate a reporter construct, the *PuRBOHF* promoter region was amplified via PCR using the specific primers listed in Table S2. The PCR product was subsequently fused into a *pGreen II 0800-LUC* vector, yielding *proPuRBOHF-LUC*. To generate an effector construct, the full-length *PuMYB169* coding sequence was inserted into a pCAMBIA1300S vector to generate a *35S-PuMYB169* fusion vector. All the constructed vectors were transformed into *Agrobacterium* strain GV1301. Mixtures or only reporters of *Agrobacterium* strains were introduced into tobacco leaves. A fluorescence assay was performed after three days using a Tanon 5200 Multi-Imaging System (Tanon, Shanghai, China). LUC/REN activity was determined using a dual-luciferase reporter assay kit (Beyotime, Jiangsu, China).

### Statistical analysis

All the data were analyzed using SPSS 17.0. Student’s two-tailed *t* test (*P* < 0.05) was used to determine differences between the two groups. The figures were generated via Origin 2019b, and phylogenetic analysis was conducted using MEGA 5.0.

## Results

### Accumulation of lignin and ROS during stone cell formation

Microscopy analysis was performed to determine the distribution and size of stone cells in the flesh of the pear fruit. As observed via light microscopy at 65 DAFB (Fig. S1), lignified stone cells were stained deep red. Most of the stone cells developed as clusters scattered irregularly throughout the fruit flesh. Secondary thickening and primary cell wall lignification are important features of pear stone cells. In the TEM images, various cell wall layers could be clearly identified because of the high lignin concentrations in those regions. As cell wall thickening progressed, the plastids and vacuoles shrank.

**Fig. 1:**
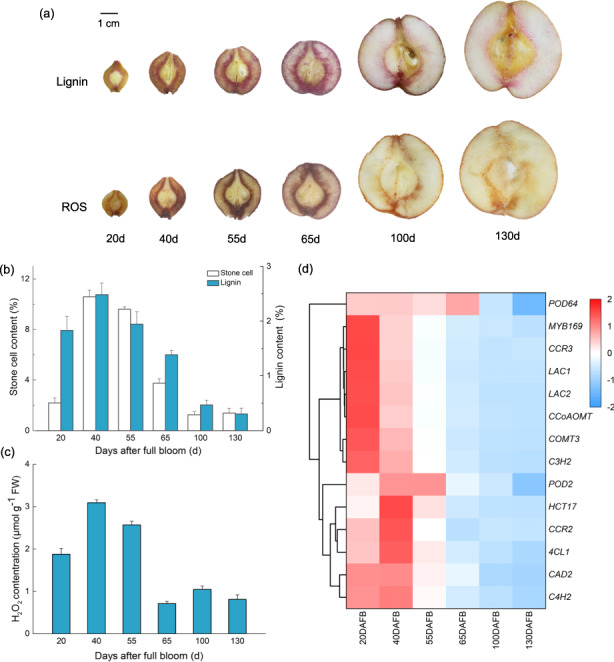
Accumulation of stone cells, lignin, and reactive oxygen species (ROS) during Nanguo pear fruit development at six developmental stages. **a** Histochemical assays of lignin and ROS. Top panel: phloroglucinol-HCl-stained sections; bottom panel: DAB-stained sections. **b** Stone cell and lignin contents. **c** Hydrogen peroxide content. Error bars: standard deviations (three replications). **d** Heatmap of the cluster analysis derived from qRT-PCR results of 14 candidate lignin biosynthesis-related genes in the fruit flesh. Red: increased expression; blue: decreased expression

To investigate the roles of ROS in the development of pear stone cells, hand-cut sections were stained with phloroglucinol-HCl and DAB. The accumulation of lignin and ROS in the samples tended to be similar, with an initial increase in the staining area followed by a decrease during the course of fruit development (Fig. 1a). In addition, stone cell, lignin and H_2_O_2_ contents were measured during fruit development. The relative contents of stone cells and lignin on a fresh-weight (FW) basis exhibited a quadratic pattern, peaking at 40 DAFB and decreasing thereafter (Fig. 1b). Similarly, the H_2_O_2_ content of the fruit exhibited a trend almost the same as that of lignin; however, the lowest H_2_O_2_ content was observed at 65 DAFB (Fig. 1c).

The expression levels of structural genes involved in the lignin biosynthesis pathway (*C4H2*, *4CL1*, *HCT17*, *C3H2*, *CCoAOMT*, *COMT3*, *CCR2*, *CCR3*, *CAD2*, *LAC1*, *LAC2*, *POD2*, and *POD64*) and related transcription factors (MYB169) were also analyzed in the flesh of Nanguo pear fruit during fruit development (Fig. 1d). Except for *CCR2*, these genes were highly expressed in the early stages and weakly expressed in later stages, which is consistent with the changes in lignin content. The expression of *PuMYB169* was similar to that of lignin biosynthesis-related genes, supporting the viewpoint that MYB169 is a transcriptional activator of lignin biosynthesis in fruit stone cells.

**Fig. 2:**
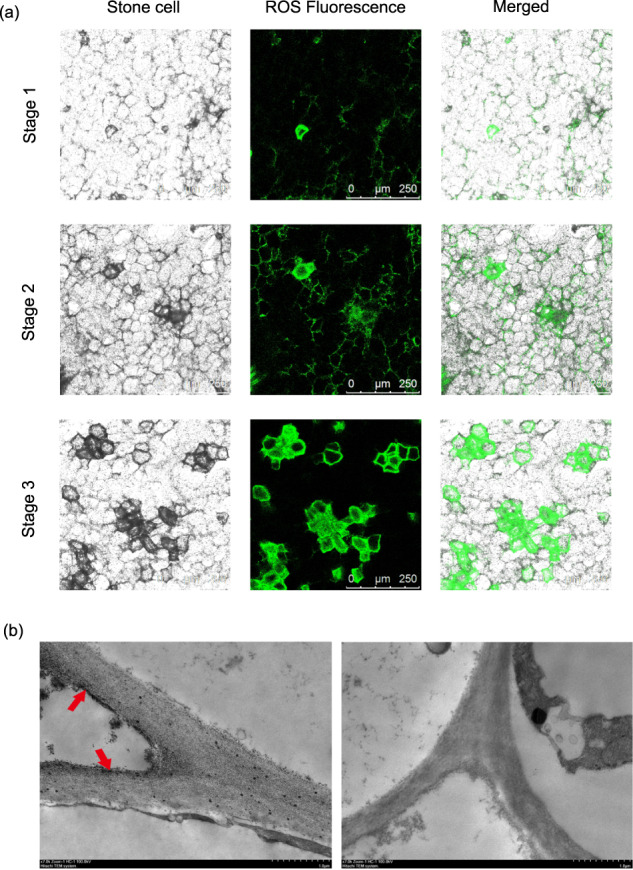
Representative images of reactive oxygen species (ROS)-localization in pear stone cells. **a** In situ detection of ROS during stone cell lignification via H_2_DCF-DA staining. H_2_DCF oxidation via ROS generates green fluorescence, whereas stone cells appear black. **b** Subcellular ROS localization, detected via electron microscopy with cerium chloride staining, which produces black cerium deposits. Left panel: cerium chloride-stained sections; right panel: control without cerium chloride

To further examine the relationship between ROS accumulation and stone cell formation, we examined H_2_DCF-DA-stained fruit sections at the cellular level via confocal microscopy. Confocal microscopy indicated strong H_2_DCF-DA fluorescence at stone cell positions (Fig. 2a), which is consistent with the ROS fluorescence positions. The intensity of the fluorescence signal increased with increasing stone cell formation. Furthermore, TEM was performed on cerium chloride-stained sections to determine the subcellular localization of ROS species during stone cell formation. There was high accumulation of H_2_O_2_ in the samples, as indicated by the obvious accumulation of black spots in the secondary cell walls of stone cells (Fig. 2b). These observations further supported the correlation between stone cell lignification and ROS accumulation.

### Involvement of ROS signaling in stone cell formation

**Fig. 3:**
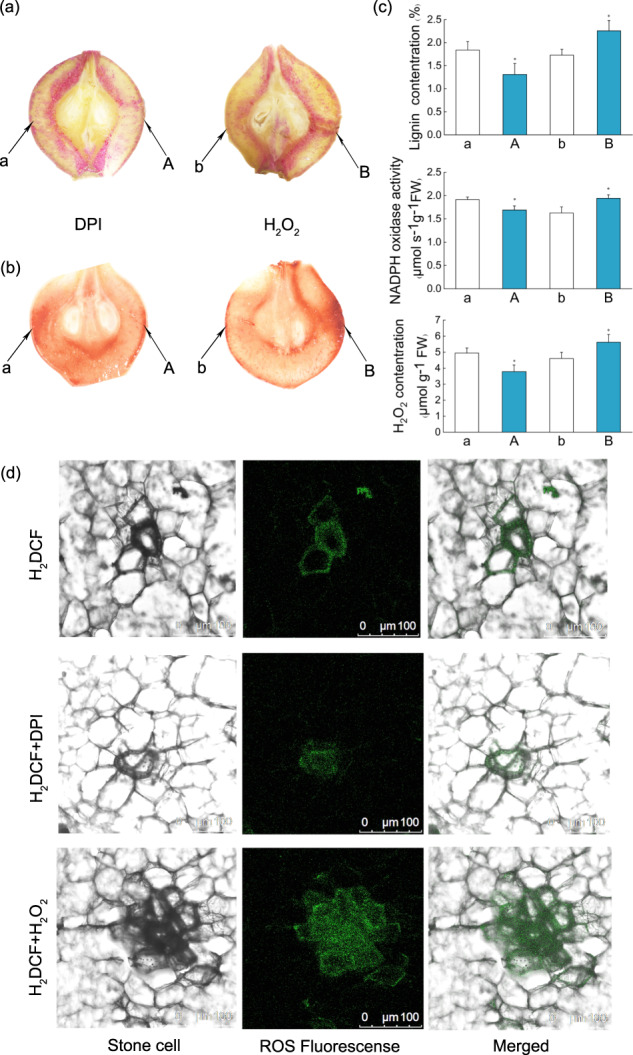
Effects of diphenyleneiodonium chloride (DPI) on stone cell formation and H_2_O_2_ accumulation in pear fruit. At 40 days after full bloom, the flesh of Nanguo pear fruit was injected with H_2_O_2_ and DPI using a syringe without a needle. Distilled water was injected as a control. Injection sites: A and B, which correspond control points a and b. **a** Phloroglucinol-HCl staining of lignin and **b** DAB staining of reactive oxygen species, observed 10 days after injection. **c** Lignin content, NADPH oxidase activity, and H_2_O_2_ content around the infiltration sites and their respective control sites. **d** H_2_DCF-DA staining of H_2_O_2_ around the infiltration and control sites. Error bars: standard deviations (three replications). *P  <  0.05

NADPH oxidase activation during defensive responses is a key enzymatic source of ROS in plants. To evaluate the role of NADPH oxidase as an ROS source during lignin biosynthesis, pear fruit were injected with H_2_O_2_ and DPI (an NADPH oxidase inhibitor) at 40 DAFB. Ten days after injection, relative to that in the control treatment, lignin staining was significantly lower at the infiltration sites in the DPI treatment and significantly higher at the infiltration sites in the H_2_O_2_ treatment (Fig. 3a). Moreover, both DAB and H_2_DCF-DA staining were performed to analyze ROS production. DAB staining revealed small quantities of ROS in the DPI treatment group but significant ROS accumulation in the H_2_O_2_ treatment group (Fig. 3b). Similarly, weaker H_2_DCF-DA fluorescence was observed at the stone cell positions in the DPI-treated samples than in the control samples (Fig. 3d).

Compared with that of the samples in the control group, the lignin content of the H_2_O_2_-treated samples was 22.13% higher, while that of DPI-treated samples was 23.39% lower. Similarly, the NADPH oxidase activity of the DPI-treated samples was significantly lower than that of the samples in the control group. Moreover, the accumulation of H_2_O_2_ in the flesh tissue of DPI-treated samples was lower than that of samples in the control group (Fig. 3d). Thus, applying an NADPH oxidase inhibitor inhibited lignin formation and H_2_O_2_ generation in the pear fruit. These data suggest that NADPH oxidase produces ROS, which plays a pivotal role in the lignification of stone cells.

### Identification of *RBOH* genes and transcripts during stone cell formation

**Fig. 4:**
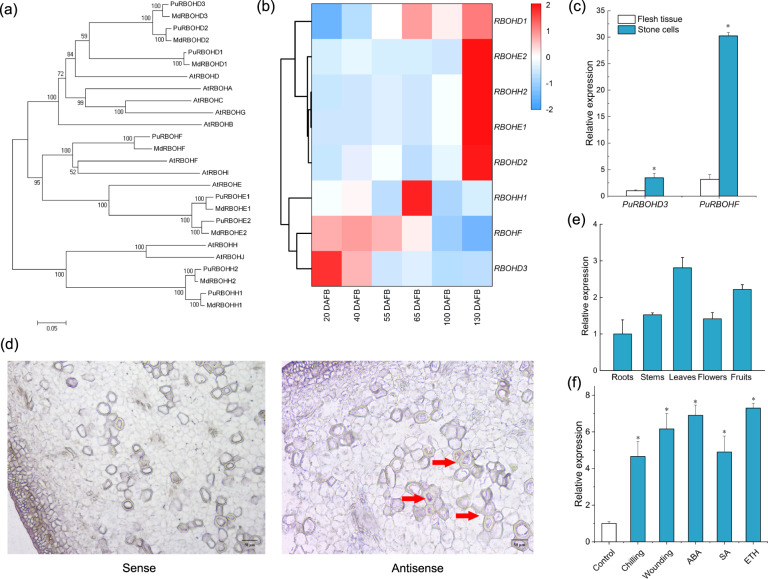
Phylogenetic relationships among RBOHs and relative *PuRBOHF* expression levels. **a** Phylogenetic tree of *RBOH* sequences of *Arabidopsis thaliana* (*AtRBOH*), *Malus domestica* (*MdRBOH*), and *Pyrus ussuriensis* (*PuRBOH*) based on a conservative approximate alignment. The scale indicates the genetic distance. **b** Heatmap of the qRT-PCR-derived cluster analysis of eight *PuRBOH* genes expressed in fruit flesh. **c** Relative transcript levels of *PuRBOHD3* and *PuRBOHF* in stone cells and fruit flesh tissues at 40 days after full bloom (DAFB). **d** RNA in situ hybridization of *PuRBOHF* in fruit flesh tissues at 40 DAFB, cross-sectioned for hybridization with sense (left column) and antisense (right column) probes. Red triangles: in situ hybridization signals of *PuRBOHF* transcripts. **e** *PuRBOHF* expression in various tissues. **f** *PuRBOHF* expression under various abiotic stresses and hormone treatments. Error bars: standard deviations (three replications). *P < 0.05

Based on similarity searches using *Arabidopsis* and *Malus RBOH* gene sequences^45,46^, eight homologous *RBOH* genes were identified from pear genomes. Phylogenetic analysis was performed to elucidate the evolutionary relationship of pear, apple, and *Arabidopsis* genes (Fig. 4a). The *PuRBOH* genes were classified into four subgroups of genes whose sequences were closely linked to the homologous sequences of *AtRBOHD*, -*E*, -*F* and -*H*.

To identify the *RBOH* genes involved in stone cell formation, we examined *RBOH* gene expression in the fruit at six developmental stages using qRT-PCR (Fig. 4b). Among the eight pear *RBOH* genes, *PuRBOHD3* and *PuRBOHF* were upregulated at the early stages of pear fruit development but expressed at low levels at the later stage, which was similar to the changes in lignin and ROS levels. To further confirm the relationships between the expression of *PuRBOHD3* and *PuRBOHF* and stone cell formation, stone cells were isolated from flesh tissues at 40 DAFB and subjected to qRT-PCR analysis. The results showed that *PuRBOHF* was expressed at the highest levels in isolated stone cells (Fig. 4c), indicating that this gene is the most likely *RBOH* member to participate in the formation of stone cells in pear.

### Expression pattern of *PuRBOHF*

To further examine the spatial expression patterns of *PuRBOHF* in pear fruit, we performed RNA in situ hybridization with a *PuRBOHF*-specific probe using flesh tissues at 40 DAFB. We found that *PuRBOHF* preferentially accumulated in the stone cell zone and found a relatively weak signal in the parenchymal cells, suggesting that this gene is involved in stone cell formation (Fig. 4d). To examine the tissue specificities of *PuRBOHF*, comparative gene expression analysis was performed using different tissues of Nanguo plants. Among these tissues, *PuRBOHF* was predominantly transcribed in the leaves and young fruits (Fig. 4e). To analyze the response pattern of *PuRBOHF* to various abiotic stresses and hormone treatments, gene expression analysis was performed using Nanguo pear fruit at 40 DAFB. Compared to their levels in the untreated fruits, *PuRBOHF* transcript levels in response to abiotic treatments (chilling and wounding) as well as ABA, SA, and ETH treatments significantly increased (Fig. 4f), suggesting that *PuRBOHF* is involved in the pear stress response. For the subcellular localization analysis of PuRBOHF, the green fluorescence signal of 35S-GFP was distributed throughout the cell, whereas the signals from the *PuRBOHF-GFP* construct were localized in the plasma membrane, indicating that PuRBOHF localized to the plasma membrane (Fig. S2).

### Transient expression of *PuRBOHF* in pear fruit

**Fig. 5:**
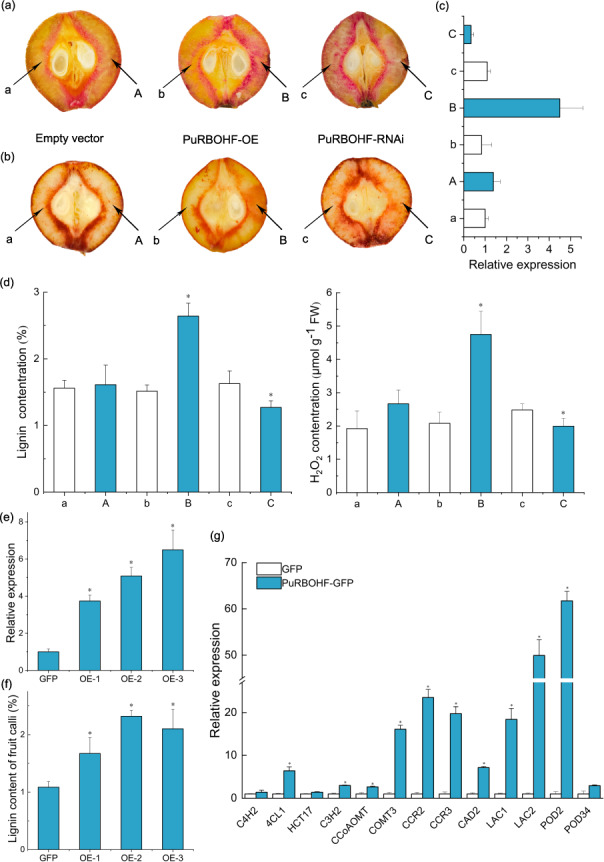
Transient expression assays verifying the function of *PuRBOHF* in Nanguo pear fruit and flesh calli. **a**–**d** *PuRBOHF* overexpression or antisense constructs were injected into the fruit flesh at 40 days after full bloom using needleless syringes. Infiltrated sites: A, B, and C; corresponding noninfiltrated sites: a, b, and c. **a** Phloroglucinol-HCl staining of lignin and **b** DAB staining of reactive oxygen species, observed 10 days after infiltration. Expression of (**c**) *PuRBOHF* and (**d**) lignin and H_2_O_2_ contents in the flesh around the infiltration sites and corresponding noninfiltrated sites. **e**–**g** Overexpression of *PuRBOHF* in pear calli. **e** Relative *PuRBOHF* transcript levels and **f** lignin contents in *PuRBOHF*-overexpressing calli. **g** Relative expression of lignin biosynthesis-related genes in transgenic *PuRBOHF*-overexpressing pear calli. Error bars: standard deviations (three replications). *P < 0.05

To further elucidate the function of *PuRBOHF* in lignin biosynthesis, *PuRBOHF* overexpression or antisense constructs were transferred into Nanguo pear fruit at 40 DAFB. Ten days after infiltration, the lignin staining was darker in the *PuRBOHF-*overexpressing samples than in the control samples (Fig. 5a). Moreover, there was a decrease in the staining intensity of the flesh at the injection site following the silencing of *PuRBOHF*. There was a 74.51% increase in the lignin content of the flesh around the infiltration sites compared with that around the noninjected sites (Fig. 5d). However, the silencing of *PuRBOHF* induced a significant decrease in lignin content. DAB-stained tissue sections were also examined to determine the effects of *PuRBOHF* overexpression or silencing on ROS generation. The amount of brown precipitates in the sites injected with the *PuRBOHF* overexpression vector was greater than that at the corresponding noninjected sites. In contrast, diluted brown precipitates were observed at the site infiltrated with the *PuRBOHF* silencing vector (Fig. 5b). The H_2_O_2_ levels were consistent with the histochemical staining results. Taken together, these results showed that the overexpression and silencing of *PuRBOHF* affected lignin accumulation in pear fruit.

### Overexpression of *PuRBOHF* in pear calli

The function of *PuRBOHF* was further confirmed by overexpressing this gene in pear calli induced from Nanguo pear flesh. Excessive lignin accumulation was observed in *PuRBOHF-*overexpressing calli compared to wild-type calli after 30 days (Fig. 5f). The transcript level of *PuRBOHF* also significantly increased in *PuRBOHF*-overexpressing calli compared with wild-type calli (Fig. 5e). Furthermore, the expression of lignin synthesis-related genes was analyzed in empty vector-transformed and *PuRBOHF*-overexpressing calli. Consistent with the increased lignin content, *PuRBOHF* overexpression also resulted in a significant increase in the transcription of monolignol biosynthesis-related genes, and *PuPOD2* and *PuLAC2* exhibited the highest increases (Fig. 5g). Thus, these genes may play key roles in *PuRBOHF*-mediated lignin biosynthesis. Taken together, these results further confirmed that *PuRBOHF* plays an essential role in lignin biosynthesis in pear fruit.

### H_2_O_2_ triggers *PuPOD2* and *PuLAC2* promoter activity

**Fig. 6:**
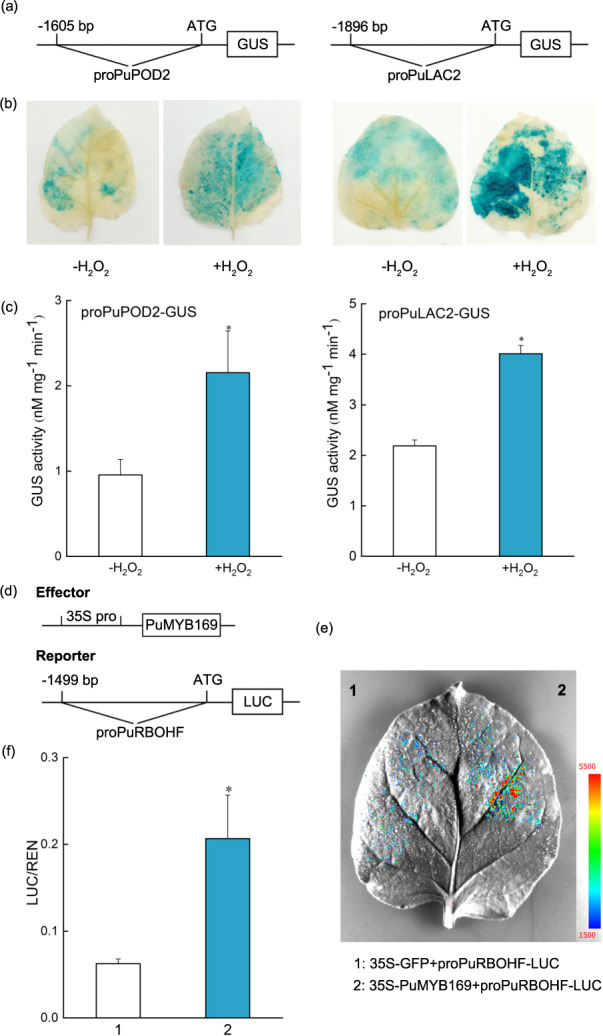
Relationship between *PuRBOHF*-mediated ROS production and the stone cell transcriptional network. **a**–**c** Effects of H_2_O_2_ on *proPuPOD2-GUS* and *proPuLAC2-GUS* promoter activity. **a** Schematics of GUS reporter gene constructs driven by the *PuPOD2* promoter or *PuLAC2* promoter. The constructs were transformed into 5-week-old tobacco leaves. The infiltrated tobacco leaves were then sprayed with H_2_O_2_ solution (+H_2_O_2_) or distilled water (−H_2_O_2_). After 3 days of growth, the leaves were harvested for **b** histochemical GUS staining and **c** GUS activity analysis. **d**–**f** PuMYB169 activated *PuRBOHF* transcription, as revealed by a dual-luciferase (LUC) assay. **d** Schematics of the reporter and effector gene constructs used in the dual LUC assay. **e** In tobacco leaves, transient expression of PuMYB169 activated *PuRBOHF* promoter activity. The transient expression assay was performed with three replicates. **f** Quantitative analysis of the luminescence intensity for each image in (**e**). Error bars: standard deviations (three replications). *P < 0.05

POD and LAC have been shown to catalyze the formation of lignin polymers using monolignols and ROS^13^. In the present study, there was an increase in the expression of *PuPOD2* and *PuLAC2* in *PuRBOHF*-overexpressing calli. To examine the effects of H_2_O_2_ on *PuPOD2* and *PuLAC2* promoter activity, *proPuPOD-GUS* and *proPuLAC2-GUS* containing GV1301 cells were infiltrated into tobacco leaves. H_2_O_2_ treatment of the infiltrated leaves caused a significant increase in GUS staining retention compared with that of leaves in the control group (Fig. 6b). Consistent with these results, enhanced GUS activity was observed after treatment with H_2_O_2_, indicating that H_2_O_2_ induced the transcription of *PuPOD2* and *PuLAC2*.

### PuMYB169 activates the *PuRBOHF* promoter

To explore whether PuMYB169 affects the transcriptional activity of the *PuRBOHF* promoter, we cloned 1499 bp of the promoter sequence upstream from the ATG codon of *PuRBOHF*, inserted this region upstream of a *LUC* reporter gene and cotransfected effector plasmids harboring the resulting *35S-PuMYB169* construct into tobacco leaves. The luciferase assays revealed that, compared with expression of the *PuRBOHF* promoter alone, coexpression of *35S-PuMYB169* and *proPuRBOHF-LUC* resulted in much stronger luminescence (Fig. 6e). These results indicated that PuMYB169 directly activated the expression of *PuRBOHF*.

## Discussion

### NADPH oxidase-mediated ROS production is essential for stone cell formation

Stone cell content and size are key factors determining the internal quality of pear fruit^47^. ROS are important signaling molecules involved in development and stress responses in plants^48^. In this study, we found that the accumulation of ROS was closely associated with stone cell lignification in pear fruit. During the fruit development period, we observed similar trends of stone cell, lignin, and H_2_O_2_ contents. Moreover, confocal microscopy and TEM imaging results further demonstrated the spatial consistency between H_2_O_2_ and lignification of the cell wall. These results indicate that ROS accumulation is closely related to stone cell development and are consistent with the findings of Heng et al.^23^. The exocarp cell H_2_O_2_ concentration was strongly positively associated with the lignin concentration.

Stone cell lignification in pear fruit was reduced by DPI. Moreover, NADPH oxidase activity and H_2_O_2_ levels were suppressed by DPI. These results further support the hypothesis that NADPH oxidase-mediated ROS production is essential for lignin biosynthesis in pear fruit. Pretreatment with DPI was shown to inhibit lignin deposition induced by cell wall damage in root tips^49^ and suppressed lignin accumulation in senescing cells in the abscission zone in *Arabidopsis*^50^. Moreover, the application of H_2_O_2_ promotes lignin formation in rice roots^22^. Taken together, these findings indicate that RBOH-mediated ROS production is responsible for stone cell lignification.

### Functional conservation and diversity of *PuRBOHF* genes

NADPH oxidases are encoded by *RBOH* genes, which are present in a wide range of plant species^51^. However, the role of RBOHs in lignification in vivo is still under investigation. *AtRBOHD* and *AtRBOHF* are closely related to lignification in *Arabidopsis*. There was a severe delay in the formation of a function Casparian strip in *RBOHF* knockdown mutants, indicating that *RBOHF* plays a role in the formation of lignin-containing Casparian strips that function as diffusion barriers in root endodermal cells^21^. Furthermore, *Arabidopsis RBOHD/F* knockdown mutants were shown to be unable to accumulate lignin in the floral abscission zone^50^. Our phylogenetic tree placed *PuRBOHF* in the same clade as *AtRBOHF*. The role of these *RBOH*s in ROS-mediated lignin accumulation in stone cells remains unclear.

It has recently been shown that *PbRBOHA* and *PbRBOHD* expression is consistent with the increased ROS content during stone cell formation in pear fruit^28^. However, there is no clear spatiotemporal evidence of RBOH involvement in the lignification of stone cells. In the present study, in situ hybridization analysis showed an increase in the expression of *PuRBOHF* in developing stone cells, indicating the involvement of *PuRBOHF* in the lignification of pear fruit stone cells. Moreover, *PuRBOHF* was localized to the plasma membrane, including sites at which lignification occurred. Therefore, we speculated that *PuRBOHF* is a putative candidate gene involved in the lignin synthesis pathway in pear fruit.

To further characterize *PuRBOHF* functions during stone cell formation, a transient transformation assay was performed on pear fruit and calli. *PuRBOHF*-overexpressing pear fruit and calli produced higher ROS and lignin contents than did fruits in the control groups. In addition, qRT-PCR analysis indicated that the expression levels of lignin biosynthesis-related genes, including *PuPOD2* and *PuLAC2*, were significantly upregulated in *PuRBOHF*-overexpressing calli. However, suppression of *PuRBOHF* expression by VIGS repressed lignin and ROS accumulation. Taken together, these results suggest that *PuRBOHF* plays an essential role in ROS formation during pear stone cell formation.

### *PuRBOHF* and the stone cell transcriptional network

ROS play a catalytic role in lignification via oxidative polymerization of monolignols to lignin^13^. H_2_O_2_ is a major ROS involved in the lignification process. In addition to its key role as a cosubstrate, H_2_O_2_ may also play a regulatory role in cell signaling^52^. The activities of PAL, C4H, and 4CL are reportedly induced by endogenous H_2_O_2_^10^. However, the relationship between H_2_O_2_ accumulation and stone cell lignification has not been previously clarified. In the present study, the expression of *PuPOD2* and *PuLAC2* was upregulated in *PuRBOHF*-overexpressing calli. Furthermore, the results of our GUS assay showed that the response of *PuPOD2* and *PuLAC2* to H_2_O_2_ was regulated at the promoter level. These results revealed that RBOH-derived H_2_O_2_ not only played a catalytic role in lignification but also induced the expression of genes regulating the polymerization of monolignols to lignin.

Xue et al.^4^ identified MYB169 as a positive regulatory transcription factor involved in lignin biosynthesis in fruit stone cells. In addition, MYB169 activates the transcription of *C3H*, *CCR*, and *CAD*. In the present study, PuMYB169 directly activated the transcription of *PuRBOHF*. Our findings thus showed that, by inducing *PuRBOHF* expression, PuMYB169 is involved in the regulation of the lignin polymerization process. However, the mechanism underlying the interaction between PuMYB169 and *PuRBOHF* requires further verification.

**Fig. 7:**
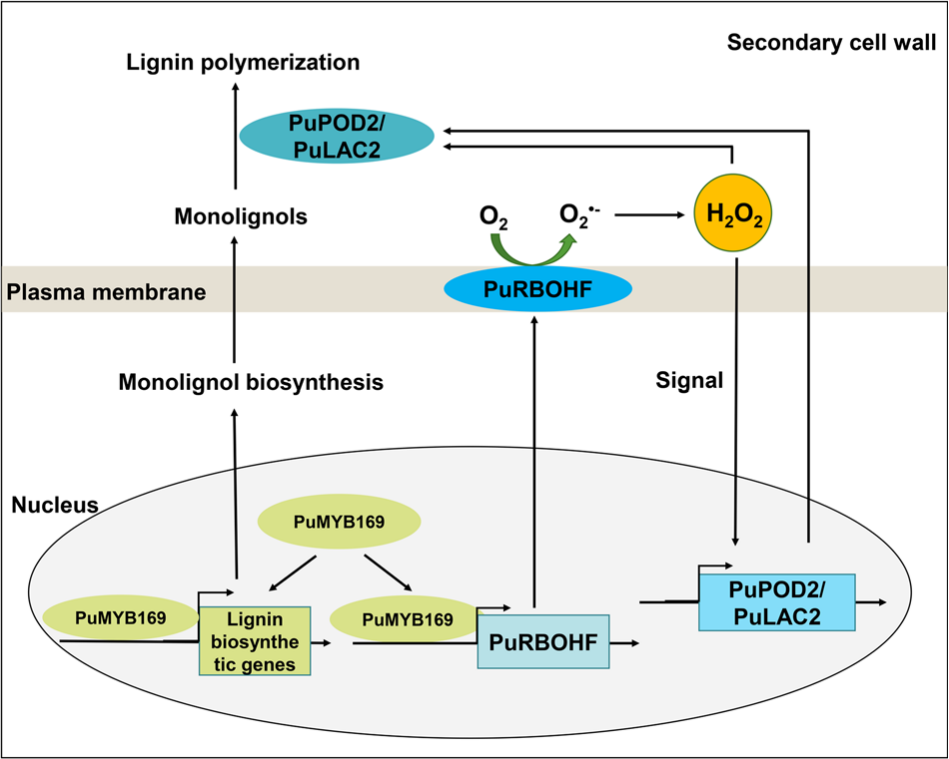
Model of the *PuRBOHF*-mediated reactive oxygen species (ROS) signaling pathway involved in stone cell lignification in pear fruit. *PuRBOHF*, controlled by PuMYB169, enhances the ROS burst in the plasma membrane. ROS then serve as secondary messengers and induce the transcription of *PuPOD2* and *PuLAC2*, ultimately promoting monolignol polymerization and stone cell formation. In addition, apoplastic ROS are necessary for the oxidative polymerization of monolignols to lignin

The presence of lignified stone cells substantially reduce fruit quality. Therefore, it is desirable to inhibit stone cell development to produce high-quality pear fruit. Previous studies have shown that ROS are involved in the lignin polymerization process^13^. The mechanism underlying the activities of ROS in lignification was elucidated in the present study. We found that, in pear fruit, stage-specific *PuRBOHF* expression contributed to a temporal peak in ROS production, which is closely associated with stone cell lignification. In turn, stone cell lignification is regulated by the upregulation of *PuRBOHF* expression, which is controlled by PuMYB169 (Fig. 7). *PuRBOHF*, in turn, induces apoplastic O_2_ production, which is subsequently spontaneously or enzymatically converted to H_2_O_2_. The H_2_O_2_ is then transported into the cytosol, where it induces *PuPOD2* and *PuLAC2* transcription. Apoplastic ROS are also necessary for the oxidative polymerization of monolignols into lignin. Overall, the findings of the present study improve the knowledge of the underlying mechanism and signaling pathways involved in the regulation of stone cell lignification in pear fruit.

## Supplementary information

Supplementary mateial

## Data Availability

Supporting data are included within the article and its supplementary information. Other relevant materials are available from the corresponding author upon request.
