# Supplementary material for: Production of reactive oxygen species by PuRBOHF is critical for stone cell development in pear fruit
Source: Hortic Res. 2021 Dec 1;8:249. doi: 10.1038/s41438-021-00674-0 (PMC8633289; doi:10.1038/s41438-021-00674-0)
Supplement: Supplementary file 1 — Supplementary mateial [file 41438_2021_674_MOESM1_ESM.docx]

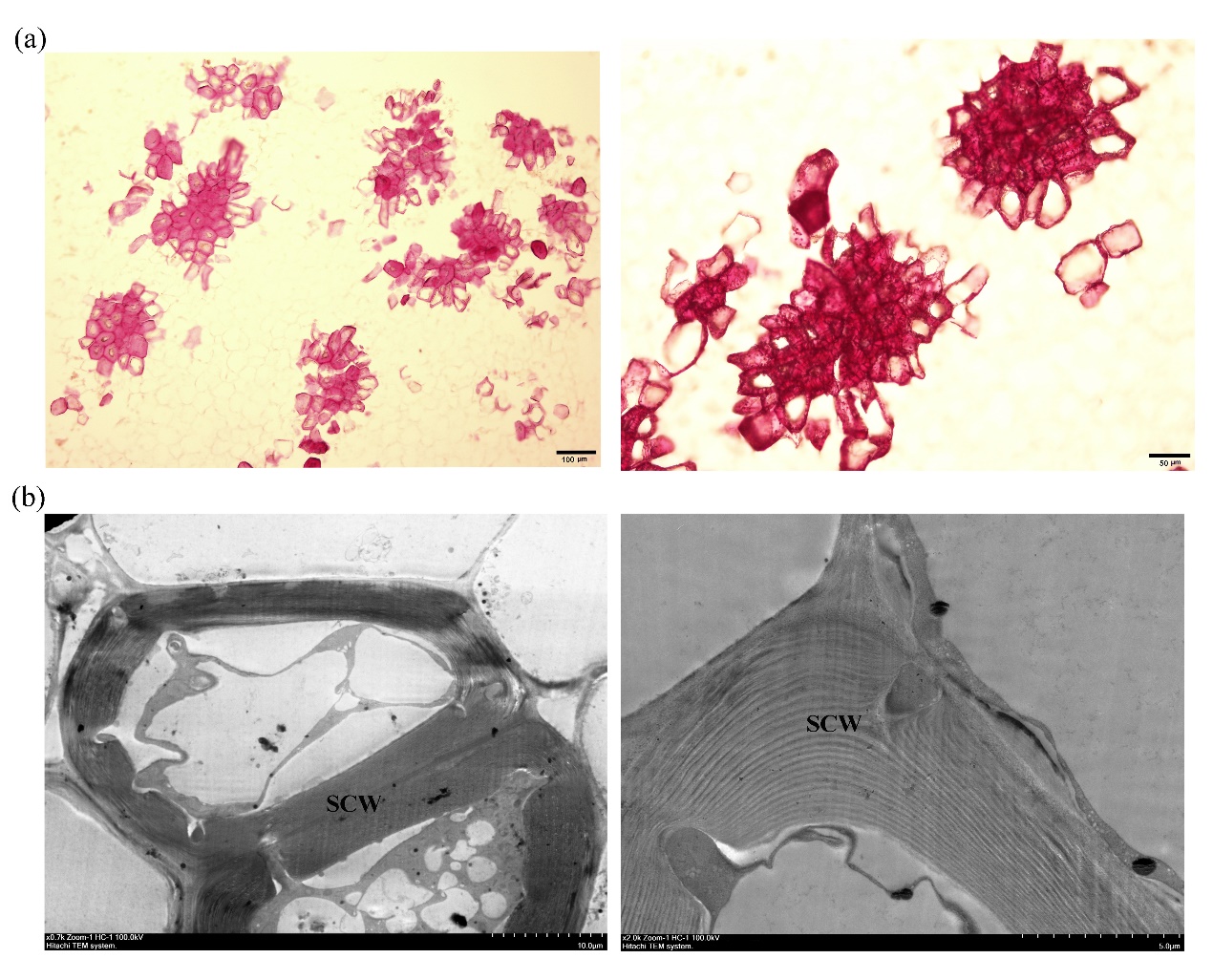


**Supplemental Figure S1** Morphological characteristics of stone cells in the ﬂesh of ‘Nanguo’ pear based on optical microscopy (a) and transmission electron microscopy (b) on 65 days after full bloom (DAFB). SCW: secondary cell wall.


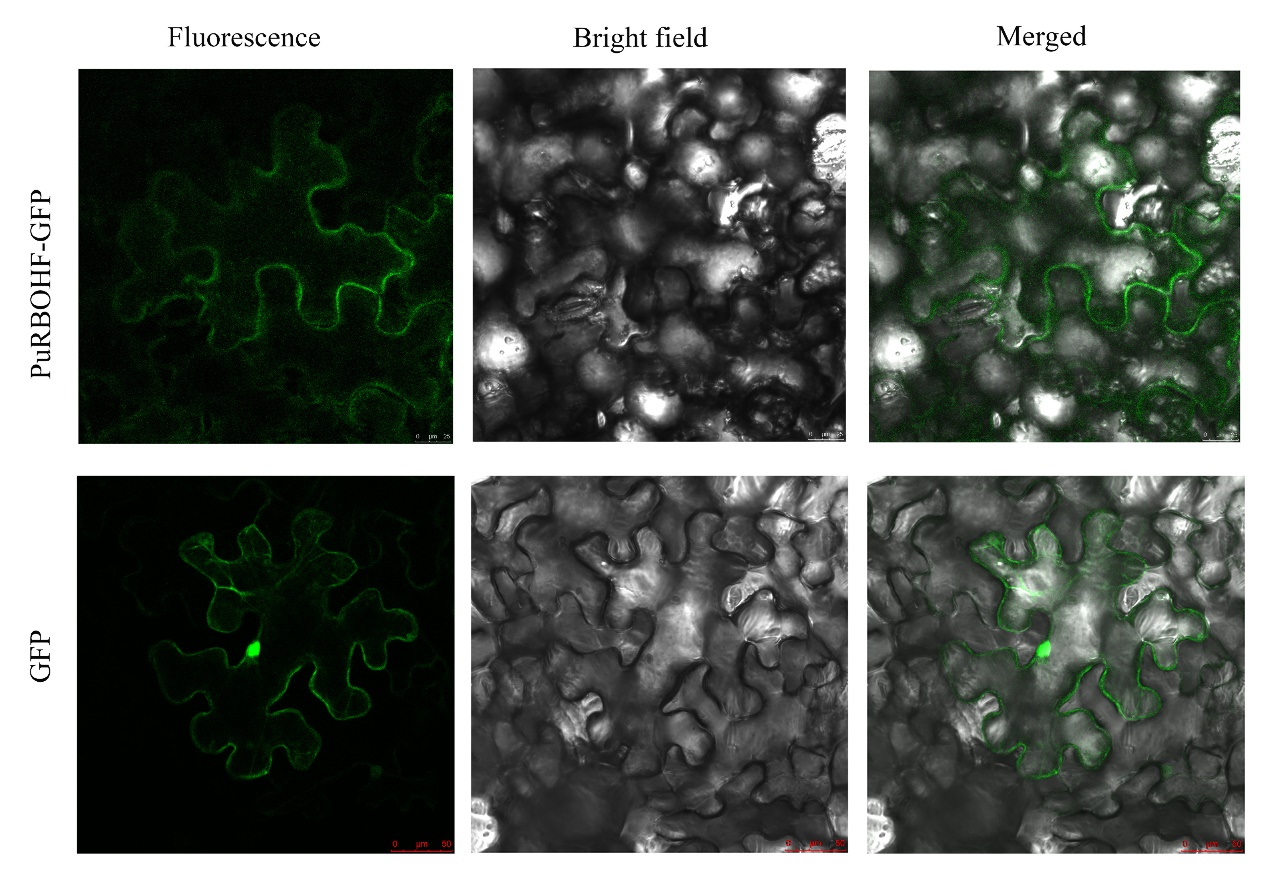
 **Supplemental Figure S2** Subcellular localization of *PuRBOHF* expressed in tobacco leaf cells.

**Supplemental Table S1** **Primers used for reverse transcription quantitative PCR analysis**

| **Gene** | **Forward primer (5′ to 3′ )** | **Reverse primer (5′ to 3′ )** |
| --- | --- | --- |
| *PuC4H2* | GCACAGCAGAAGGGAGAAATC | GTCGGGCTCTGTGATTTGTAC |
| *Pu4CL1* | CATGCTCCTCCTCCAAAACAG | TCCTTCACCTTGTCCACGTAA |
| *PuHCT17* | TTCAGCAACCTCATTCCCTCT | AAGGTCATCAAGATTGCCACG |
| *PuC3H2* | ATTGTGTCACTTCTGCAGGAC | AAGGGAGGCTGGAGAAATCAA |
| *PuCCoAOMT* | GGAAAGATCTTGGCCATGGAC | TAGTTGTCTTTGTCAGCGTCC |
| *PuCOMT3* | AAAAAACACAGAGCAGCAATGGCAG | TGGTGGTTCATTTGATTCGACGGCAGAG |
| *PuCCR2* | CGTCCGGAAACAAAGCTAATAAACA | GGTTCCTCTAACAGTGTAGCCTCTTTCG |
| *PuCCR3* | CTAATAACCAAGATGCCTGCCGATA | GGTTCCTCTAACAGTGTAGCCTCTTTCG |
| *PuCAD2* | AAGGAAACTGAGGAGATGCTTGAAT | TACTTTATTAAATAAGATTGCTGCCG |
| *PuLAC1* | GGCAAGACCTATCTGCTACGAA | GGGCATTGGTGGTTTGTCC |
| *PuLAC2* | ATCCAGGCGTCGTTCCAAA | GGTGTCCGTCTTGAAGGGTTT |
| *PuPOD2* | TCATCTTATTCTAGTGTGGTC | GGCGTAGTAGTTTGTTTTG |
| *PuPOD34* | GCCACTTCAGGTAAAGAT | ATAGAAAGGAAGCAGCAAT |
| *PuRBOHD1* | TGCCGGAACACCATAACCTT | AGCTCGTAGTCATCGCTGTT |
| *PuRBOHD2* | AACATGGCCTTGATCCTGCT | AATCGCACGTCAAATGAGCC |
| *PuRBOHD3* | GGAACAAATCTCCGACGAAA | CCATGATGTAGCCGACACTTG |
| *PuRBOHE1* | GAAACACGCTGACATGCCTT | TTTTCCGGCGAAGAGTTTACC |
| *PuRBOHE2* | TACCCGGAAACGTGATTAGC | GCTATGGCTCGCTTCAATTC |
| *PuRBOHF* | TGGCGTTTTCCTCTATCTCGT | TGTGGCTTCGACATTTGCAA |
| *PuRBOHH1* | GAGCAGGCTTCCTTTGAATG | TAGCCAAGTCGGAAAACACC |
| *PuRBOHH2* | AGGGGAACTCTTACCGCAAT | TGTCCCAGCAACATCCATGA |
| *PuActin* | CCATCCAGGCTGTTCTCTC | GCAAGGTCCAGACGAAGG |

**Supplemental Ta****ble S2 Primers used for vector construction.** The restriction sites are underlined.

| **Name** | **Forward/** **Reverse primer** **Sequence (5′ to 3′ )** | **Description** |
| --- | --- | --- |
| pC1300S-PuMYB169 F | GGTACCATGGGAAGGCAACCGTGC | Subclone to pC1300S Vector of PuMYB169 |
| pC1300S-PuMYB169 R | CTGCAGCTACAAAAGCCCATATGCCCA | Subclone to pC1300S Vector of PuMYB169 |
| pFGC5941-PuRBOHF 1F | CCATGGCTCGCTCCGGCGCTGAGAAA | Subclone to pFGC5941 Vector of PuRBOHF |
| pFGC5941-PuRBOHF 1R | ATTTAAATTCGAATCGAAACTTTGATCGGAAATTTGGGA | Subclone to pFGC5941 Vector of PuRBOHF |
| pFGC5941-PuRBOHF 2F | GGATCCTCGAATCGAAACTTTGATCGGAAATTTGGGA | Subclone to pFGC5941 Vector of PuRBOHF |
| pFGC5941-PuRBOHF 2R | CCCGGGCTCGCTCCGGCGCTGAGAAA | Subclone to pFGC5941 Vector of PuRBOHF |
| pC1301S-PuRBOHF 1F | CATTTGGAGAGGACAGGGTACCCGGGGATCCATGAGGGC  TCATCCGAGGCACGA | Subclone to pC1301S Vector of PuRBOHF |
| pC1301S-PuRBOHF 1R | ATCACAAGCGAGATGGTT | Subclone to pC1301S Vector of PuRBOHF |
| pC1301S-PuRBOHF 2F | AACCATCTCGCTTGTGAT | Subclone to pC1301S Vector of PuRBOHF |
| pC1301S -PuRBOHF 2R | GGTGAACAGCTCCTCGCCCTTGCTGTCGACAAAGTGTTCC  TTGTGGAATTCGAAC | Subclone to pC1301S Vector of PuRBOHF |
| LUC-PuRBOHF F | CTGCAGTGATGAATAAAACAGAATGATAATTAACAA | Subclone to PGreen-LUC Vector of PuRBOHF promoter |
| LUC-PuRBOHF R | GGATCCACCATAATGAAAGCCTGAATTGAA | Subclone to PGreen-LUC Vector of PuRBOHF promoter |
| GUS-PuLAC2 F | GTCGACAAGACTGAATTCCAACCAAATTGA | Subclone to PBI101-GUS Vector of PuLAC promoter |
| GUS -PuLAC2 R | GGATCCCATGTAGCAAGAACAACGTTGTTAAC | Subclone to PBI101-GUS Vector of PuLAC promoter |
| GUS -PuPOD2 F | GTCGACAGACAACAAATAGAGCGAGAGAGTCA | Subclone to PBI101-GUS Vector of PuPOD promoter |
| GUS -PuPOD2 R | GGATCCTTTCTTAGTGACAGCATCTTTGACAA | Subclone to PBI101-GUS Vector of PuPOD promoter |

**Supplemental** **File S1.** The coding sequence of *PuRBOHF* and promoter sequences of *PuRBOHF, PuPOD2, PuLAC2*.

**Coding sequence of *PuRBOHF***

ATGAGGGCTCATCCGAGGCACGAGCGCCGATGGGCTTCGGACTCCGTCCCAGAGAGGTCCATGGTCAGCGACGGATCATCATCTCCGGCAATGACCGACGACTCCAACTCCAGGGATCAGGAGTTCGTCGAGGTAACTCTCGACTTACAAGATGACAACACGATTGTCCTCCGCAGCGTCGAGCCCACCACCGTCATTCACATCGATGACCTCACAGGCGCCGGAAATGAAACTCCGACGTTGGCTTCAGCTTCTTGGTCGCCATCCCCGATACGGAGAAGCTCGTCCAACAATCGGATACGGCAATTCTCTCAGGAGCTCAAAGCAGAGGCCTTGGCCAAAGCGAAGCAATTTTCACAAGAGCTCAAGGCCGAGCTGAGGAGGTTTTCGTGGAGCCAAAGCCACAGCTCGAGAGTTCTCTCCGCTTCAACTTCTCAGAATGCATCAGCCGGTGGCGGCGGAGGCGATGCGTTTGACTTGATGCTGGCCGCTCGCGCTCTGAGGCGGCAGCGAGCACAGCTCGATCGCACTCGCTCCGGCGCTGAGAAAGCGCTTCGGGGATTAAGGTTCATTGGTAACTATAATAGTACGAAGACCAATGGCGTCGATGCTTGGAACGAAGTGGAGACCAATTTCAACAAGCTCACCAAAGACGGCCATCTCTTCCGCGCAGATTTCCCTCAATGCATCGGAATGAGAGATTCGAAGGAGTTTGCGTTGGAGTTGTTCGATGCGTTGGGTCGAAGACGCAGACTGAAGGTTGAGAAAATCAGCAAGGACGAGCTCTATGAGTTTTGGTCCCAAATTTCCGATCAAAGTTTCGATTCGAGGCTCCAGATCTTCTTCGACATGGTGGACAAGAATGACGACGGTCGAATTACAGAAGAAGAAGTAAAAGAGATCATCATGTTGAGTGCTTCTGCAAACAAGCTATCAAGATTGAAGGAACAAGCAGAGGAATATGCAGCTTTGATCATGGAAGAGTTGGATCCTGAAAGACTTGGCTACATTGAGTTATGGCAATTGGAGACGCTTCTACTACAAAAAGATACGTACTTAAACTACAGCCAAGCACTAAGCTACACGAGCCAAGCCCTAAGCCAGAGCCTCCAAGGGCTGAGAAGGAGAAGTCCAATACGCAAAATGAGCACCAACTTAATCTATTATTTGCAGGAAAATTGGAGGAGAGTCTGGATTTTGACATTGTGGTTTTGCATTATGATTGCGCTCTTCACATGGAAATTCATACAGTACAAGCAAAAAAGTGCTTTTCAGATAATGGGTTATTGCCTTCTCACGGCCAAAGGTGCTGGCGAGACCTTGAAATTCAACATGGCTCTTGTGCTGTTGCCTGTTTGTAGAAACACCATCACTCGGCTCAGGTCCACCAGGCTTGGCTTCTTTGTGCCTTTTGACGACAACATCAACTTCCACAAGACAATTGCTGCAGCCATTGTAGTTGGTGTCATTCTCCATGCTGGGAACCATCTCGCTTGTGATTTTCCGAGGCTTATAAAGGTTTCCGAATCAGTCTACAAGAAGTATTTGCTTCACGACTTTGGAAAGCATAAACCCAGCTATCTAGATTTGATTAAAGGGCCAGAGGGGCTGACTGGAATTATTATGTTGATATGCATGATTATTGCTTTCACACTCGCTACACGGTGGTTCAGGCGTAACCTCGTTAAGCTTCCCAAGCCCTTCAATAGGCTCACTGGCTTCAATGCCTTCTGGTATTCACATCACTTGTTTGTTATTGTCTACGTCTTGCTCATTATCCATGGCGTTTTCCTCTATCTCGTGCACACGTGGTACCTAAAGACGACTTGGATGTATCTTTCTGTTCCTGTTTTACTATATGCTGGAGAAAGAATCTTGAGAATCTTCCGCTCTGGCTTCTATACCGTCCGTCTTCTGAAGGTTGCTATTTATCCTGGAAACGTTCTCACATTGCAAATGTCGAAGCCACAACAGTTTAAGTACAAGAGTGGACAGTACATGTTTGTACAGTGCCCAGCAGTTTCTCCGTTTGAGTGGCATCCATTTTCGATTACATCTGCTCCCGGTGATGACTACCTTAGTGTTCACATTCGTCAGCTTGGTGACTGGACGCAAGAGCTTAAGAGGGTTTTCTCAGAAGCTTGTGAGCCCTCCTTAGCTGGGAAGAGTGGTCTTCTAAGGGCTGATGAGACAACCAAGACAAGTTTGCCCAAGCTTTTAATAGATGGGCCTTATGGTGCCCCAGCCCAAGACTACCGGAATTATGATGTCCTGCTGCTTGTTGGACTTGGAATCGGTGCAACGCCATTCATCAGTATTTTAAAAGATCTGCTCAACAATATTGTCAAGATGGAAGAGCAGGCAGATTCTGTTTCAGATTTCATTAGAACATCAGAACTAAGTTCTGGGAGTATAGATTCTCCCAATCCCAACAAGGTTTATCCAAAACGAAAAAAGCCACTGAAGACCACTAATGCCTACTTTTATTGGGTAACAAGGGAGCAAGGCTCTTTTGATTGGTTCAAAGGAGTGATGAATGAAGTTGCAGAGCTTGATCAAAGGGGTGTCATTGAGATGCACAACTACTTGACTAGTGTGTACGAGGAAGGTGATGCTCGATCAACACTCATCACCATGGTTCAAGCTCTCAACCATGCTAAGAATGGGGTGGACATTGTGTCTGGCACAAGGGTGCGGACCCATTTTGCAAGGCCTAATTGGAAGAAAGTTTTCTCAAAAACTTCCTCCAAACACTCTAATGCAAGGATAGGGGTATTCTATTGTGGAGCACCAGTTTTGGCAAAAGAACTCAGCCAGCTTTGCTATGACTTCAATCAAAAAGGTTCAACCAAGTTCGAATTCCACAAGGAACACTTTTAG

**Promoter sequence of *PuRBOHF***

TGATGAATAAAACAGAATGATAATTAACAAACAGAAACAGTTGTGTATAATTTAAAAATGAATGGAGGGAGGTGGTGGTGTTTATAAGCGTTGTAAAAATGCAGAGGTGAGGAGAGTGATGAGTGAGTTTTGTTTTCTTCTATTCTTTTTGTGAAAAGAGAGTGAATTGGTCGTGGAACGCGGTTTGTTCGGAATCTCAGGGAAAAGACATGTACGCGGAACGCAGCATTAGACAGAGAAAAAAGAGAGAAGCAGACAAAGCCTTGTATTCAGAGAAGGAAGAAAGCTAAAAGTACTCAATTATCTCGTGGGGAGCATACAATAAAACCACCACAATTATATTATACATGTACATATTTATGTGTTATATATGTACGTTTATTTATTTATTTTATGTAAATTGATATTTGTTATGAAATTATATGCGGATGTGGTTACGGTTTGTTTGAATGTGTTACACGCAAGGATTCAAAAGGTGCTAGGCACTGGTTGGGCGACGGATATGTGTCTAGGTGGGAGTTTAGGCAGAGCCTAGGTGGGGGTTTAGGCAGTTTTTTTTTTTTAATTTTAATTGAATTTATTATATAACATATAAATAACTGCCTACTTGTACTTAAAAAAATACATCATTGTATTAAAACGCTTGAATTGCAAAATAGAATGATATATTGATTATAAAATATTCAAACATATTGAAAATATGAGAAACAACATACAATGAGTGCTCATCCAAACATTCAAGAAATCTCGTACAATTTAATGAAAAAATAAAATGCAAGATGGAAGTTATCTTTCTTAAGTAAGAATTTCGACCTAAGTGGGTGGACAGGTCTAGGTGGGCGCCTAGGTAGGCTAGGTGCCCTTTCTTAATTTTAAATGCTTAGGGACTAATCATGTCGGTAACCAGCCGCCTAGCACTAGGCAGAGATTTTTAGAACACTAGTTACATGCATTATTTGTAATGGTGACTTATTATTTGAATGTCAAGCTTTTATGCATTATCAATTGTGTTTTTATATCCCGCAAACTGAGAATTTAGCTTGAGTAGTTATTTTAGTTTTATTCGTCATCTAACAGTGTTGAGAAATTTGGAAAAATAACTAAATTTTAAATCCCGTATAGAATGTTAACGATCATTTTAATTTTATGTAATTATGTAATTATAATCAATACTTGATGTAAAAGTATTAATATACCCTTAATATTAGGATTTCTGACAACAACCAAAACACAACCTTTAAACTACCTTAAAATGAGGAAATAATCTATATCACTTTTTAGTTTTCATTATTCTCTTCAACTTTTGAACTACTTTCTGCAACCTTTCGGATTTTTGCAAAACGATACAACAGCCTTATTGTTACCTTTGACTGCACTTTCATTCTTCAATCACATCAATTTTAAAATCTTATTGGAAGTACCGAGATACCTAGCAATTTAATCTCGAAGACTTTGTTTTTTTTATTCTTTTTTGTTTCAATTCAGGCTTTCATTATGGT

**Promoter sequence of *PuPOD2***

AGACAACAAATAGAGCGAGAGAGTCAAAGTTGAAATGATGATTACTAACCTTGTCTCTCATTCAAAATTGTGCATTAGACGTTCATCTTGCAAGACTCCCAAATGATAAAAGAAAGAACTCATTTTTGTTTATTACCTTCATCACATATGTACAAGACCAAATTCATTTGATTTCTAAAAAGAATAAGCGAAACGCAGTAGCTAGCTGTCTGCTTTTGAGTTGAGTAGTAAGAGTCAGAAAGCAATATCAGTCATTCTTAGAATTAGCATTCTATAACCCAAGACTCGAGTGGACAAGAGAAAAAAGCTCTTCATGATCCATTTGTAGGTCGATTTTCTGAAACCACAAAATTCTTATTTAGAATGAGATTTCAACTAAAAGAAAATTGATATATTCACCTATAGTATAGATAATTGTTTTCTGTAATCACTAGGCATAGACATTATATATCATTGTCTATATCCGATAGATAATGCATAATTCCATTTGTTTTATTATCTACCTAGAATTAATTTGGGAAGGCACTAGCAATTTAGTCAGTTCTTATCCACAAACCGGGTCCATCTTGGTTGGCCATTCCCCATCATCTTAATCATATAGACAATACTTAGTTGTCAATCTGTTAAAAAGCTCACGTTTTTAACCTTAAACAAACAAAAAAAAACTTTAAATTATGCATAACAAATTACAAACCCTATTGTCATGAGTCATGTTTTATAACACTGGATCTATGCCAAGTGGCTGGTCCATTACACTATTCTGGTATAGAAAGCCACAGCTAGGCCACAGGAACCTTAACGTCCAGTCTACGATCTAGTTGTGTCTACTTTAGCAGCCTCCAAATCCACTTTTAGACCACGTTTTCATCTTTTCTCAAATCATTTTTATATAAGAAAACTAAAGAAAAATGTTTAAAAATTTTGAATTTTAACGATAAGAATAAAATAAAGGGTAACGTAAATAGTACCAAAATTGATTTTTTTGTATAAAAAATGTGATTTTTCGTTAAAATAAACAGTACCGGTAACTTTTCGTTAAAATTCCTTTTTTATATTAGGGTTGTTACCACGTATTTAGATTAAAATCACTATATTTTATTATTTTAGTGCAAACTCAAACATTCGGACAAAGTTGCAGTGCAGTAAGAAGAAGGTAAACAGGGGCGGCAACTGAAAATTTGACAGGCCCAAATCAATGTCTCTTTCGAACACACGATAACTAGTCTACGTTCAAATGCATTTGCTTTAATATGAACCTACTTGTCTATATTAAAAAAAGTAAAGATTCCTCAATCCAACTGGCCCATATAGACTATAGACTTAAGCGCCCTCTTCTTTTTATATATTCCTCCACCCTACCATTCAAAATCTCAGTCTGCTTCTTCTTCTCTCTCTCTCTCTCTCTCTCTCTCTCTCTTCATATCTGTGTTCTGAAAAAATAAAATTTTTGGTCGAATGGGTCGATTTCATCTTATTCTAGTATGGTCACTTTGCGTCAGTTTGTGTCTACTCCTTTGTCCCACATCGGCGCAGCTCAAAACAAACTACTATGCCAATATCTGCCCCAACGTTGAAAGCATTGTCAAAGATGCTGTCACTAAGAAA

**Promoter sequence of *PuLAC2***

ATGATGAAGACTGAATTCCAACCAAATTGACCATCATAACCTCTAATCACCGTACACATGTTAATTTGTCAAGTACGCTTAATGTAATCAGTCATGTGTTTGTGTGGGAGGATGAGTAGGTTTTTTTATTAAAAAAAGGGGAAATAATATGGACAAAGCAAAGAAGTTGATGAAAAAAAAAAGAGCATGCTGAAGAGGGAGTTTGAAAAAGACTGGGTGACCTACCGACCTTCGGTTTAAATTTGATTGGCTTTCAAACAGACTATCATGTCGTCCATCGTCCATCTGTTGGAGCTCCTATCTGATATATGACCTGAAAGTCATTTCTCAACCATAAGCTAATTTGTTCCCTAAGCATCCAATTAGGCGGCATGCAGTAGATGTTTAGCATTTTATACTTCCCTATACGCATTTCACTACCTACCAGATCTACATGTATTTGGCTTCTCACTTCCACTTCAATTAAGATGAAATGCCTAAGCATGCATCCAATTAAGATGAAATACCAAATACGTATGCCCTTATTTCTGCAATTAGTTAATAGCTTAAAAATGTCAAAAAAATCTCCCAAAATCTTATCGGTCCATTGGGATGTAGCTTGATGGTAATCGATCACGTCATGTTTGTAGTGTTTGTATCATTTTCATAACATACTCGATATTTTAACAGATTGTGTCGTATCAAATCCATTCAAATAAACAGGTAATACAACCCAACCCAAACTTGACCCGTTAACATTAACGGGTAATATGACTTGGTTATTAACTTAAGCAAGAGTACTATTTGCTAGCGGAGCTTCTTTGGGAATGTCATTGTTTTTCTTATTTTAAAATATCGGGAATGAGAGCATCTCGGCATTACATTTACATATTATTTTGCATTTTCGACGTATATAAATGTGAATTTTACTCCAATGGATGAATAAAGTTGGGAACTCATTTATTATCCGACCAACAAGAAATGTAAAATGCTTATTTCAAGCCATTTTGCACTCTGGTAATTGAAAATTTGTTCTAATGTTTTGGTTTGTCTATAGAGTAGCACTGAAAACAAAAAATCCGTTCACAATAATGCAGCAATGTTTAATTCATTTAAATTTAGGGCTAGTTTAAATATTACATGTTATTGACATATACACTTACACATTATTTGGTAAACAAATTTAATAAAAAAAAATGGTGACTCTAGTATTGATACCTTTATTTAGGTTCATAATTATGCTAAAACATGTATAAGAAATTTGGTATTAACTACTTTGCTTCCAAATATACTCAAAATTATTACTAATGAATGGATAAAAAACCAAACCTCACTAATATAAGCATATCCAAATGGTCCACCAGCTGCTGGGTTGACCCATATACTTAATGTATAAGTTGTCCCAAAACACTCAACAAAGTCCACCCGATTAACCAAATTAGTAACAAAAAGGATTAAATCAGATAAAGGGAAATTTAATAAGCATATTATAAACAGCATTTACC.GAAATATGCAGCTGGAAGCTGGTAAATTAGAAACTTTATGTAACATGTAAGAAATCACAGGGAAGACCTTGGGTTAGCTTAAGAATCTTGTTTTCATAGATTACCATCGTCCACCTACCCTGGAAAAACGACATCACAGCCGTGCCTCTTGTCACCCTTTATAAGCTTGCGCTGGCAAGTCATGACCATGAACCCAACACCAATAGCAACAAAAACCATTAAGAAACGAAGTTTATCGTACTAGTACACTAGCTAGTGCAAAGAGAATCAGAGAGAGTATTGAGAAATGGTGTGGTCGATTCGATTTCTTGTGCTTCTTCTGGCATGTGTTCTTCCTGCTTTGGTTGAGAGCAGAGTCCGACACTACAAGTTTAATGTAAGTACCGTTAACAACGTTGTTCTTGCTACATG
